# A rare clinical image of sacral double crush syndrome with S1-S2 Tarlov cyst and L5-S1 disc disease

**DOI:** 10.11604/pamj.2025.52.99.49443

**Published:** 2025-11-05

**Authors:** Gurjeet Kaur, Nikita Gangwani

**Affiliations:** 1 Department of Musculoskeletal Physiotherapy, Ravi Nair Physiotherapy College, Datta Meghe Institute of Higher Education and Research (DU), Sawangi, Meghe, Maharashtra, Wardha, India

**Keywords:** Tarlov cyst, perineural cyst, disc bulge, spine magnetic resonance imaging, radiculopathy, double crush syndrome

## Image in medicine

Sacral double crush syndrome is an uncommon neurological condition characterized by simultaneous compression of sacral nerve roots at multiple anatomical sites. The prevalence of symptomatic Tarlov cysts ranges from 1-5% of all identified perineural cysts, while concurrent presentation with lumbar disc pathology is exceptionally rare. A 52-year-old male presented with chronic diffuse buttock pain progressing over 2 years. Initial mild gluteal aching gradually intensified, affecting daily activities and developing into neurological symptoms, including right lower limb weakness, paresthesia during prolonged standing, and difficulty walking. Acute exacerbation occurred following heavy lifting, manifesting as sudden, sharp radiating pain from the lumbar spine to the right lower extremity. Physical examination revealed positive sciatic tension and flexion abduction external rotation (FABER) signs with altered sensation in S1-S2 dermatomes. Motor examination demonstrated weakness in the gluteus maximus, hamstrings, and gastrocnemius muscles. Distinctive clinical findings included spinal extension aggravating L5-S1 disc-related symptoms, while flexion worsened Tarlov cyst manifestations. Magnetic resonance imaging indicated a prominently positioned Tarlov cyst at the S1-S2 level, concurrent with L5-S1 disc space narrowing and posterior disc bulge. This radiological combination created a double crush phenomenon, explaining the complex overlapping symptomatology affecting the S1 nerve root territory. The case emphasizes the importance of comprehensive imaging evaluation for complex spinal pathologies affecting multiple neural structures.

**Figure 1:**
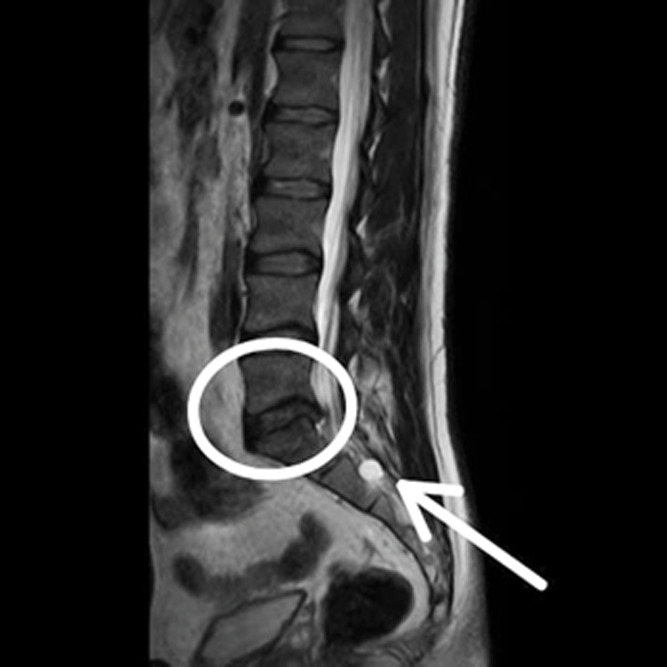
sagittal T2-weighted magnetic resonance imaging demonstrating a prominent sacral Tarlov cyst at the S1-S2 level with concurrent L5-S1 disc space narrowing and posterior disc bulge, T2 hyperintensity noted in the cerebrospinal fluid-filled perineural cyst, degenerative changes with disc height loss at the L5-S1 level, S/O double crush phenomenon affecting the S1 nerve root territory

